# Nanozymes: An emerging arsenal for the treatment of *Candida albicans* infection

**DOI:** 10.1016/j.fmre.2024.11.021

**Published:** 2025-01-09

**Authors:** Yanni Song, Mengyuan Chang, Heng Dong, Ning Li, Guisheng Zeng, Yue Wang, Dongliang Yang

**Affiliations:** a Key Laboratory of Flexible Electronics (KLOFE) and Institute of Advanced Materials (IAM), School of Physical and Mathematical Sciences, Nanjing Tech University, Nanjing 211816, China; b Nanjing Stomatological Hospital, Affiliated Hospital of Medical School, Institute of Stomatology, Nanjing University, Nanjing 210008, China; c Fujian Key Laboratory of Drug Target Discovery and Structural and Functional Research, School of Pharmacy, Fujian Medical University, Fuzhou 350122, China; d A*STAR Infectious Diseases Labs (A*STAR ID Labs), Agency for Science, Technology and Research (A*STAR) 138648, Singapore; e Department of Biochemistry, Yong Loo Lin School of Medicine, National University of Singapore, 117597, Singapore

**Keywords:** *Candida albicans*, Nanozyme, Fungal infection, Catalytic therapy, Combination therapy

## Abstract

*Candida albicans* infections, exacerbated by the emergence of drug resistance and biofilm formation, pose a significant threat to human health and challenge current treatment options. Therefore, it is imperative to develop novel and effective therapeutic strategies against this pathogen. Recently, nanozyme-based catalytic therapy (NBCT) has emerged as a promising tool against fungal infections, as these enzyme-like catalytic materials exhibit strong antifungal activity against drug-resistant pathogens. Compared to traditional therapies, NBCT offers broad-spectrum antifungal activity and rarely induces drug resistance. Additionally, NBCT can be combined with other treatment modalities to enhance antifungal efficacy. Research efforts are being expedited to make NBCT applicable for treating various forms of *Candida* infections. In this perspective paper, we summarize recent progress in NBCT and its combination strategies in the treatment of these infections. Finally, the challenges and prospects of NBCT are discussed.

## 1 Introduction

The yeast *Candida albicans* (*C. albicans*) is a commensal organism in the normal human microbiota, commonly inhabiting the skin, oral cavity, and gastrointestinal tract [1]. However, when the microbiota is disturbed or the host immune system is compromised, *C. albicans* can transition from a commensal to a pathogenic state, leading to life-threatening infections [2]. Clinical data reveal that *C. albicans* is the primary cause of candidiasis. According to reports from various countries, *C. albicans* contributes to 38% to 70% of invasive candidiasis cases and directly causes nearly 40% of related deaths [3]. *C. albicans* can cause a broad range of infections in various tissues, including meningitis, oropharyngeal candidiasis, endophthalmitis, endocarditis, keratitis, cutaneous candidiasis, esophagitis, urinary tract infections, vaginitis, and other diseases [4]. In addition, *C. albicans* is a significant contributor to clinical device-associated infections. Currently, the treatment of candidiasis mainly relies on three classes of antifungal drugs: azoles, echinocandins, and polyenes [5]. However, prolonged and uncontrolled use of antifungal drugs can result in the emergence of drug resistance, primarily through mechanisms such as increased expression of drug efflux pumps, mutations in drug targets, or alterations in membrane permeability [5,6]. Moreover, *C. albicans* can also form biofilms, where fungal cells are embedded in a dense extracellular matrix, which provides strong resistance to antifungal drugs and the host's immune system, often resulting in frequent treatment failure [7]. Therefore, there is an urgent need to develop novel antifungal strategies to tackle the challenges posed by candidiasis.

**Fig. 1:**
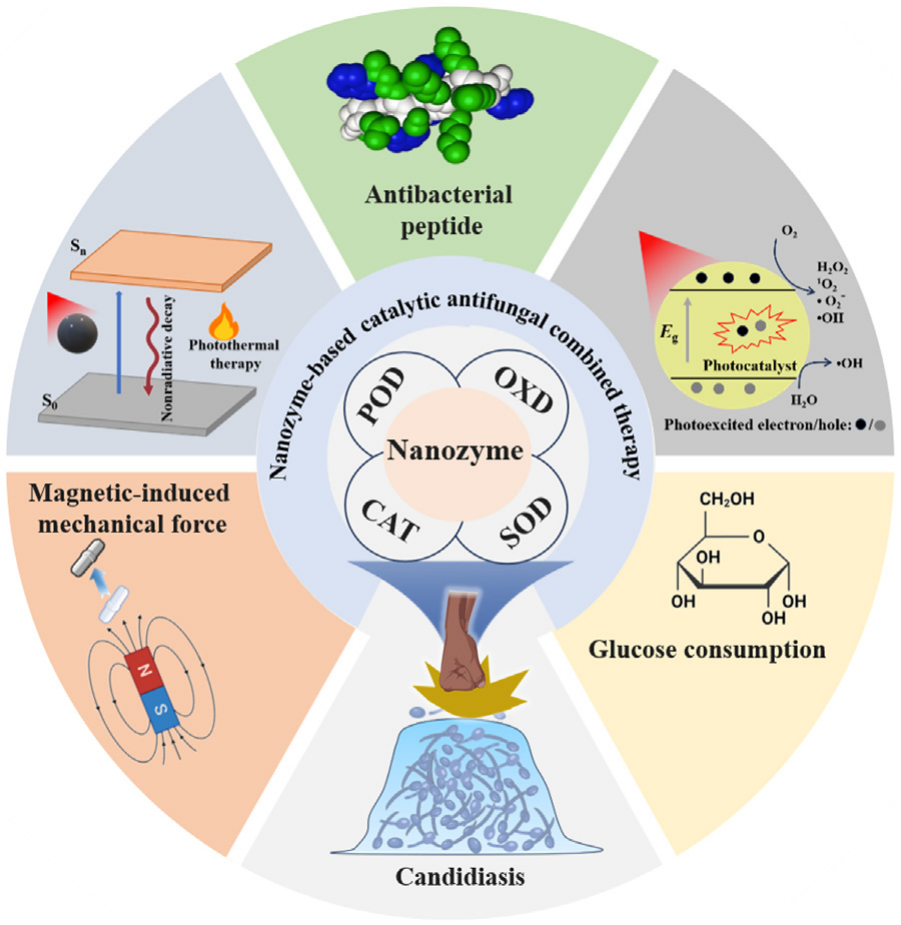
**NBCT for the treatment of candidiasis**.

Nanozymes are a class of nanomaterials with enzyme-like activity [8]. Compared to traditional natural enzymes, nanozymes offer advantages such as strong catalytic activity, good stability, and low cost, making them a promising substitute for natural enzymes [9]. So far, researchers have synthesized a variety of nanozymes with peroxidase (POD), oxidase (OXD), haloperoxidase, and catalase (CAT)-like activity [10,11]. These nanozymes include carbon-based, noble metal, transition metal, and organic-inorganic hybrid nanomaterials [10]. Some nanozymes can catalyze the production of reactive oxygen species (ROS), destroy microbial components, and inhibit or kill fungal cells. As a result, they have garnered considerable attention for their potential in the treatment of fungal infections. In addition, some nanozymes have photothermal, photodynamic, and magnetothermal properties [12]. Therefore, nanozyme-based reagents have shown great promise as therapeutic agents for candidiasis. In this perspective paper, we describe the characteristics and benefits of nanozymes and nanozyme-based catalytic therapy (NBCT) and summarize their progress in developing novel therapeutics for candidiasis (Fig. 1). At the end, we will discuss the challenges and future directions of nanozymes in the treatment of *Candida* infections.

## 2 NBCT for candidiasis management

Some nanozymes can mimic the catalytic activities of natural enzymes to catalyze the production of ROS, disrupting the fungal redox system and causing fungal death [13,14]. Recently, Hui Wei's team utilized hyaluronic acid (HA)-based hydrogel to embed POD-like nanozymes (rGO@FeS_2_) and *Lactobacillus* for the treatment of mouse vaginal candidiasis [15]. The HA-based hydrogel can be degraded by hyaluronidase secreted by *Candida*, releasing rGO@FeS_2_ and *Lactobacillus*. The probiotic *Lactobacillus* grows in the vagina and secretes acidic metabolites and hydrogen peroxide (H_2_O_2_). In an acidic environment, rGO@FeS_2,_ with POD-like activity, breaks down H_2_O_2_ to produce hydroxyl radicals that kill *Candida* cells without affecting the activity of *Lactobacillus*, thereby managing vaginal candidiasis. In the treatment of keratitis, eliminating infectious *Candida* cells and alleviating excessive inflammatory response can accelerate healing. Based on this idea, Zhu's team synthesized manganese oxide nanocluster-decorated graphdiyne nanosheets (MnO_x_/GDY) with OXD, POD, superoxide dismutase (SOD), and CAT-like activities. They loaded these nanosheets into HA and polymethyl methacrylate-based microneedles to penetrate ocular and fungal biofilm barriers [16]. MnO_x_/GDY can exert OXD and POD-like activities in the biofilm environment, converting oxygen and H_2_O into ROS to eliminate *Candida* and control infection. Simultaneously, the SOD and CAT-like activities of MnO_x_/GDY can be activated in the inflammatory area, clearing excessive ROS and alleviating hypoxia and inflammation, and further accelerating the repair of infected tissues.

To enhance the efficacy of nanozyme-based catalytic antifungal therapy, combining NBCT with other antifungal strategies, such as phototherapy, antimicrobial peptides, and physical therapies, has attracted significant attention. The development of nanozymes with photocatalytic and photothermal activities can augment ROS generation. For example, hyperthermia can accelerate the catalytic reaction rates of nanozymes, such as POD-like and OXD-like catalytic activities, and enhance pathogen susceptibility to ROS. Additionally, photocatalysis offers an alternative pathway for ROS production. Both strategies can increase ROS levels, thereby enhancing the therapeutic efficacy of ROS-based antifungal treatments [17,18]. The development of antifungal peptide-functionalized nanozymes enhances the stability of peptides and improves the interaction between nanozymes and pathogens [19]. Utilizing antifungal peptides to disrupt the cell membrane of *C. albicans* potentiates the antifungal effect of ROS-mediated catalytic therapy [20,21]. For example, in the study by Yuan et al. [21], heptapeptides (amino acid sequence: IHIHICI) self-assemble with nickel ions to form β-helix nanotubes (Ni-IH-7). Ni-IH-7 exhibits both the antifungal activity of antimicrobial peptides and enzyme-like (POD and phospholipase C) catalytic activity. After incubation with *Candida*, Ni-IH-7 can bind to the fungal surface, destroy the cell wall, and cause fungal lipid peroxidation, resulting in fungal ferroptosis. Approximately 95% of *C. albicans* are inactivated by Ni-IH-7 nanotubes within 10 min.

Physical fungicidal strategies, such as mechanical force therapies, have also garnered attention for their ability to kill pathogenic fungi without inducing drug resistance [22]. For instance, ferro-based magnetic nanomaterials with nanozyme activity have been synthesized and investigated [23,24]. To achieve self-supply of H_2_O_2_ substrate, Zhou et al. synthesized glucose oxidase-functionalized magnetic nanoparticles [23]. These nanoparticles could deprive fungal cells of glucose, as glucose oxidase breaks down glucose, resulting in the production of gluconic acid and H_2_O_2_. Under the catalysis of a POD-like iron-based nanozyme, H_2_O_2_ is converted into hydroxyl radicals that cause damage to *C. albicans*. Meanwhile, the mechanical force generated by magnetic nanoparticles within an external magnetic field can destroy the extracellular matrix of *C. albicans* biofilm and eventually eliminate the entire biofilm. In another research conducted by Koo's team, they precisely controlled the magnetic field using a programmable algorithm to regulate the aggregate shape and the movement of magnetic nanoparticles [24]. This magnetically induced mechanical force enabled the peeling of *C. albicans* biofilm on the surfaces of implants and catheters. At the same time, POD-like magnetic nanoparticles catalyzed H_2_O_2_ to produce hydroxyl free radicals to inactivate *C. albicans*, adding in the treatment of candidiasis. Therefore, by disrupting fungal redox homeostasis and damaging fungal cell components and structures, the application of NBCT and NBCT-based multimodal therapies can eliminate drug-resistant *Candida* and regulate the microenvironment at the infection site, offering a novel solution for treating *Candida* infections. With advancements in nanotechnology and increased research on nanozyme-based antifungal reagents, nanozymes hold immense potential for future clinical applications. The current nanozymes for *C. albicans* removal are summarized in Table 1.

**Table 1:** **Overview of nanozyme-based antifungal therapy**.

Material type	Enzyme-like activity	Anti-fungal activity	Infection model	Antifungal mechanism	Ref
rGO@FeS_2_	POD	Yeast elimination	Mouse vaginal candidiasis	Hydroxyl radical	[15]
MnO_x_/GDY	OXD, POD, SOD, CAT	Yeast elimination, antibiofilm	Mouse keratitis	Hydroxyl radical, H_2_O_2_, and other ROS	[16]
Copper- and iodine-doped carbon dots	POD	Yeast elimination, antibiofilm	Mouse wound and vaginal candidiasis	Hydroxyl radical, photocatalysis	[17]
Copper sulfide nanoparticles	POD	Yeast elimination	Mouse cutaneous fungal infection	Hydroxyl radical, antimicrobial peptides	[20]
Ni-IH-7 nanotube	POD, phospholipase C-like activity	Yeast elimination	Mouse vaginal candidiasis	Hydroxyl radical, antimicrobial peptides	[21]
Magnetic nanoparticles	POD	Yeast elimination, antibiofilm	In vitro	Hydroxyl radical, glucose consumption, magnetic-induced mechanical force	[23]
Iron oxide nanoparticles	POD	Yeast elimination, biofilm removal	Human gingival spheroid	Hydroxyl radical, magnetic-induced mechanical force	[24]

## 3 Challenges and perspectives

As a novel antifungal strategy, NBCT offers distinct advantages, including low dose requirements, high efficiency, and selective catalysis of specific substrates at the infection site. Compared with antimicrobial peptides, metal ions, quaternary ammonium salts, and phototherapy, NBCT has excellent stability and high biosafety, with its antifungal activity unaffected by light penetration depth [21,[25], [26], [27]]. The inherent physicochemical characteristics of nanozymes allow their catalytic activity to be regulated by local physicochemical factors, such as the redox level and pH. In addition to the utilization of endogenous stimulators to regulate nanozyme activity, exogenous stimulators, such as heat, light, magnetic field, and ultrasound can also be employed to regulate catalytic performance. In practical applications, researchers and medical staff can regulate catalytic activity flexibly based on patients' needs. Despite the progress made in using nanozymes to treat fungal infections, this field is still in its nascent stage, and some challenges remain to be addressed.

1. Thoroughly investigating the catalytic principle of nanozymes and elucidating the relationship between the catalytic mechanism of nanozymes (*i.e.*, catalytic activity, selectivity) and structure-activity parameters (*i.e.*, size, composition, morphology, and surface properties). This will provide theoretical support for linking clinical efficacy and enzyme structure.
2. Fully understanding the specific mechanism of *C. albicans* removal by nanozymes. Analyzing the specific mode of action can offer guidance for the design of nanozymes in subsequent stages.
3. Developing non-invasive imaging techniques and specific probes for real-time monitoring. The complex tissue environment within the body presents a significant challenge for monitoring the activity of nanozymes. Hence, non-invasive imaging techniques, such as photoacoustic imaging, magnetic resonance imaging, near-infrared two-region photoacoustic imaging, and specific probes of catalytic products or substrates can facilitate the investigation of the catalytic activity of nanozymes *in vivo*.
4. Expanding the combination of NBCT with other treatment modalities to achieve synergistic antifungal therapy and enhance its effectiveness. Combination therapy generally reduces the dosage of drugs and mitigates the toxic side effects of therapeutic agents. Additionally, employing different antifungal mechanisms helps to curb the development of *C. albicans* resistance.
5. Achieving large-scale production of nanozymes and controlling the preparation of high-quality nanozymes. This is a critical issue that needs to be addressed to facilitate subsequent industrial transformation or clinical application. For example, iron oxide nanomaterials, as FDA-approved magnetic contrast agents, have been widely utilized in clinical applications [28]. As a POD nanozyme or a magnetically activated mechanical therapeutic agent, iron oxide nanomaterials have demonstrated a satisfactory anti-*C. albicans* biofilm effect. Therefore, we believe that the NBCT may hold significant economic value in the future.
6. Utilizing clinically approved components to expedite the clinical transformation process during the design and synthesis of nanozymes. In terms of material components, developing nanozymes with single components that exhibit high enzyme activity represents a promising trend for future development [29].
7. Systematically evaluating the safety of nanozyme-based antifungal agents *in vivo*. It is crucial to monitor the safety of nanozyme metabolism *in vivo* and the effects of catalytic substrates produced at targeted and non-targeted sites on normal tissues and physiological processes. Additionally, the effects of the final components of metabolizable nanozymes on the body and the long-term toxicity of non-biodegradable nanozymes accumulated in the body need to be assessed.
8. Investigating the relationship between antifungal nanozymes and fungal virulence factors. Currently, the antifungal activity of nanozymes is primarily mediated by oxidoreductase, which uses ROS levels to control and eliminate *C. albicans*. However, there are few reports on antifungal nanozymes that directly target fungal cell wall components.
9. To effectively deliver nanozymes to subcutaneous infection sites, surface functional modification, such as attaching polyethylene glycol or CD47 to nanozyme surfaces and coating nanozymes with cell membranes extracted from patients, can enhance efficient nanozyme accumulation at the infection site, effectively reduce non-specific protein adsorption, and avoid capture by the mononuclear phagocytic cell system [30]. Additionally, this strategy prolongs the circulation of nanozymes *in vivo*.

## Declaration of competing interest

The authors declare that they have no conflict of interest.
